# On-demand zero-drag hydrodynamic cloaks resolve D'Alembert paradox in viscous potential flows

**DOI:** 10.1038/s41378-024-00824-z

**Published:** 2024-12-12

**Authors:** Neng-Zhi Yao, Bin Wang, Hao Wang, Chen-Long Wu, Tien-Mo Shih, Xuesheng Wang

**Affiliations:** 1 School of Mechanical and Power Engineering, East China University of Science and Technology, Shanghai, 200237 China; 2 Department of Mechanical Engineering, University of California, Berkeley, CA 94720 USA

**Keywords:** Nanofluidics, Nanopores

## Abstract

The possibility of freely manipulating flow in accordance with humans will remain indispensable for breakthroughs in fields such as microfluidics, nanoengineering, and biomedicines, as well as for realizing zero-drag hydrodynamics, which is essential for alleviating the global energy crisis. However, persistent challenges arise from the D’Alembert paradox and the unresolved Navier-Stokes solutions, known as the Millennium Problem. These obstacles also complicate the development of hydrodynamic zero-drag cloaks across diverse Reynolds numbers. Our research introduces a paradigm for such cloaks, relying exclusively on isotropic and homogeneous viscosity. Through experimental and numerical validations, our cloaks exhibit zero-drag properties, effectively resolving the D’Alembert paradox in viscous potential flows. Moreover, they possess the capability to activate or deactivate hydrodynamic concealment at will. Our analysis emphasizes the critical role of vorticity manipulation in realizing cloaking effects and drag-reduction technology. Therefore, controlling vorticity emerges as a pivotal aspect for future active hydrodynamic zero-drag cloak designs. In conclusion, our study challenges the prevailing belief in the impossibility of zero drag, offering valuable insights into invisibility characteristics in fluid mechanics with implications for microfluidics, biofluidics demanding the drug release or biomolecules transportation accurately and timely, and hypervelocity technologies.

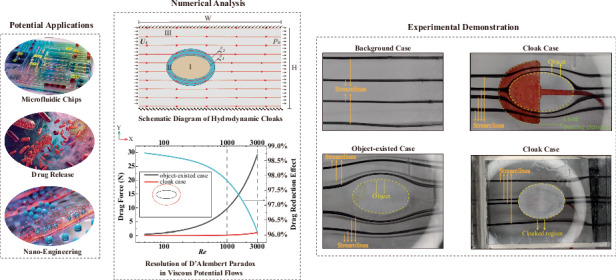

## Introduction

Invisibility characteristics, which ensure interference-free interactions between the intrusive objects and peripheral environments, are of great importance in modern microfluidics and nanoengineering, such as biomolecules transported in microfluidic chips or biomedicines manipulation, including accurate drug release^1,2^. Likewise, invisibility contributes to achieving hydrodynamic zero-drag performance by eliminating mutual interactions, playing a vital role in addressing the escalating global energy crisis that arises from the ever-increasing demand for energy worldwide. For energy crisis alleviation, one of the critical challenges lies in overcoming drag during the motion of objects. Consequently, the urgency to advance drag-reduction technology^3–5^, particularly in marine transportation, the automotive industry, aviation, military, pipeline systems, and biofluidic and microfluidic communities, has become imminent. In conventional methods, researchers primarily focus on superhydrophobic surfaces^6,7^, bioinspired texture surfaces^8,9^, fluid-infused surfaces^10,11^, and others. However, these methods do not allow the resistance against the motion of an object to be completely eliminated.

Inspired by electromagnetic cloaks^12,13^ that can hide the objects electromagnetically by regulating the path of the electromagnetic wave, hydrodynamic cloaks^14–18^ and thermal cloaks^19–23^ have been developed. These hydrodynamic cloaks are capable of reducing the drag on objects moving in a fluid. Besides, they can redirect fluid flows while maintaining the original distributions of the external hydrodynamic fields where blunt-body flows occur. Therefore, they have been investigated profoundly, and have attracted extensive attention.

The concept of hydrodynamic cloaks was first proposed to study porous media^14,15^ for creeping flows and laminar flows on the basis of Darcy’s law and transformation theory^12,13^, which regulates the fluid flows through the manipulations of permeability. Subsequent experimental studies^24–26^ are conducted successfully to validate the effectiveness of transformation media under the flow control and have provided valuable insights into the practical realization of hydrodynamic cloaks at the experimental level.

For the purpose of developing the transformation theory in hydrodynamics beyond porous media, transformation hydrodynamics^16^ is first proposed by manipulating the fluid viscosity in creeping flows and further extended to laminar flows^27^. Hereafter, a multitude of hydrodynamic meta-devices based on transformation hydrodynamics, such as hydrodynamic concentrators^28^ and hydrodynamic rotators^29^ have emerged. Additionally, the proposal of arbitrary space transformation theory^30–32^ has further expanded the achievement of hydrodynamic cloaks and enhanced our comprehension of space transformation. Nonetheless, the inhomogeneous and anisotropic parameters of these cloaks proceed to challenge experimental fabrications.

To address these issues, Tay et al. have attained a metamaterial-free hydrodynamic cloak by virtue of the scattering cancellation method^33^. Furthermore, innovative methods such as convection-diffusion-balance theory^34^, electro-osmosis method^35^, deep-reinforcement-learning approach^36^, and meta-hydrodynamics theory^37^ have been proposed. More comprehensive studies regarding hydrodynamic cloaks can be found in the review^38–41^. However, previous studies have seldom explored the drag-reduction properties of hydrodynamic cloaks, and even fewer have considered the potential for achieving zero-drag performance or the underlying mechanisms driving this phenomenon.

To overcome these challenges, here we propose a paradigm to fabricate hydrodynamic zero-drag cloaks with the analytical solution. By verifying our hydrodynamic cloaks experimentally and numerically, we find that the elliptical hydrodynamic cloaks exhibit zero-drag characteristics over a large-*Re* range. Furthermore, these characteristics of the cloak illustrate that we resolve the D’Alembert paradox^42,43^ in viscous potential flows, which had been proven to exist only in the ideal fluids. Moreover, the hydrodynamic cloaks can switch on and off at will, which significantly enhances the accurate flow manipulations. Finally, we discover that the vorticity transport explicitly determines the drag-reduction and cloaking effects of hydrodynamic cloaks, and hence propose that the vorticity controlling probably becomes a promising perspective to achieve hydrodynamic zero-drag cloaks under higher *Re*’s.

## Theoretical design

**Fig. 1:**
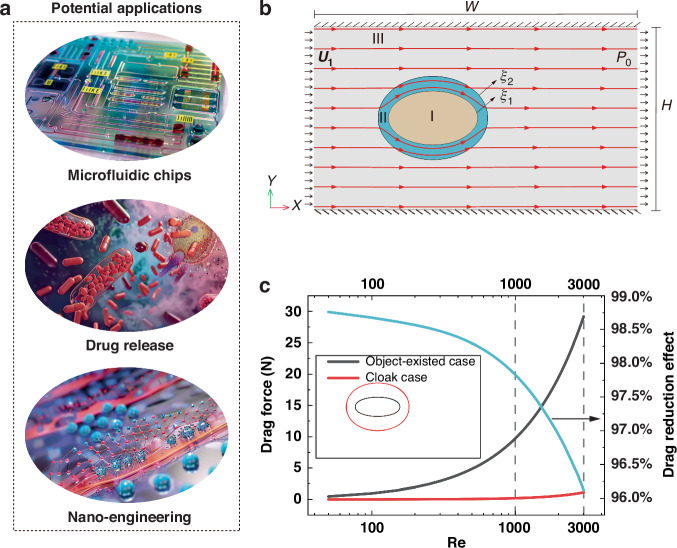
Potential applications, schematic diagram, and zero-drag characteristics of the proposed hydrodynamic cloaks. **a** Potential application of hydrodynamic zero-drag cloaks upon mutual hydrodynamic interactions between intrusive objects and peripheral environments are eliminated. **b** Two-dimensional model of elliptical hydrodynamic cloaks, where ξ_1_ and ξ_2_ indicate the geometric parameters of the object (region I) and cloaks (region II), and both of them are horizontally located, steadily immersed in a freestream (region III). **c** Drag of the cloak wrapped around the object (red line), drag of the object existence only (black line), and drag-reduction effects (cyan line) vary with various *Re*’s. Hydrodynamic cloaks that can be switched on and off at will are seen in the supplemental video

According to Newton’s Third Law, free interferences imply zero drag on the objects in motion through a fluid. For example, precise biomolecule transportation and stable drug release (Fig. 1a) can be ensured by eliminating mutual interferences (equivalent to zero drag) between transported objects and microfluidic chips or biological systems, thus lowering the energy input, minimizing the rejection rates and achieving sustained release effect. Additionally, the ability to switch the hydrodynamic cloak on and off will enable targeted drug delivery in biological systems. Similarly, in the case of nanoengineering research (Fig. 1a), such as intracellular measurement^1^, free interferences provided by hydrodynamic metamaterials promise more accurate manipulation just parallel to electromagnetic metamaterials^2^. Therefore, it is imperative to design hydrodynamic cloaks capable of entirely mitigating interferences between moving objects and the surrounding fluids, as well as achieving zero-drag characteristics.

The governing equations of continuity and momentum transport for steady-state incompressible flows without the influence of body forces can be expressed as

1 $$\begin{array}{rcl}&&\nabla \cdot {\boldsymbol{u}}=0,\\ &&\rho {\boldsymbol{u}}\cdot \nabla {\boldsymbol{u}}+\nabla p=\mu {\nabla }^{2}{\boldsymbol{u}},\end{array}$$

where ***u*** and *p* are velocity vector and pressure, respectively. Symbols *ρ* and *μ* denote density and dynamic viscosity, respectively.

To solve the analytical solution of the elliptical hydrodynamic cloaks (Fig. 1b), the solution of Navier-Stokes equations believed one of the Millennium Problems has to be settled first, which in turn challenges the design of hydrodynamic zero-drag cloaks^44,45^. Therefore, simplifications of Navier-Stokes equations become necessary. It is commonly known that the vorticity aggravates the instability of fluid flows, namely, elimination of vorticity emerges as one promising perspective for accurate flow control. Hence, we focus on viscous potential flows considered as irrotational flows, in which Navier-Stokes equations can be transformed to Laplace-like equations. Ulteriorly, the analytical solutions of Navier-Stokes equations and hydrodynamic cloaks can be obtained using variables separation method^46^ (see Supplemental Material S.1). Accordingly, the dynamic viscosity of elliptical hydrodynamic cloaks can be expressed as

2 $${\mu }_{c}=\frac{-\coth ({\xi }_{2})+\coth ({\xi }_{1})}{-\tanh ({\xi }_{2})+\coth ({\xi }_{1})}{\mu }_{b},$$

where *μ*_*b*_ and *μ*_*c*_ denote the dynamic viscosity in the background (region III in Fig. 1b) and hydrodynamic cloak (region II in Fig. 1b).

Apparently, dynamic viscosity merely relates to geometry parameters and dynamic viscosity of the background, which indicates constant. It is worth mentioning that although our cloaks are predicated on viscous potential flows, they can hold firmly in any real fluid flow insofar as vorticity is eliminated and featuring irrationality patterns. In summary, the paradigm for homogeneous and isotropic hydrodynamic zero-drag cloaks is established, and the analytical solution for elliptical hydrodynamic zero-drag cloaks is obtained as well.

## Results and discussion

To verify the effectiveness of the proposed paradigm, experiments and numerical analysis are conducted. For hydrodynamic cloak manipulations, numerous flow-control technologies abound, such as bionics^47,48^, optofluidics^49,50^, magnetohydrodynamics^51,52^, electroosmotic flows^53,54^, thermo-hydrodynamics^55,56^, and among others. Here, we choose the easy-to-implement thermostatically controlled method (belonging to thermo-hydrodynamics), which matches the viscosity manipulations more accurately (see Supplemental Material S.2).

To construct irrotational flows required by our paradigm, a classic viscous potential flow named Hele-Shaw flows^57,58^, that exhibit irrotationality, is involved to validate the elliptical hydrodynamic cloaks experimentally and numerically. Computational simulations are conducted using commercial software COMSOL Multiphysics. The geometrical sizes of computational model are *H*_height_ × *W*_width_ × *D*_depth_ = 600 mm × 300 mm × 50 µm that patterns Hele-Shaw flows^57,58^. The confocal length of both elliptical objects with *ξ*_1_ = 0.45 and hydrodynamic cloaks with *ξ*_2_ = 1 is *f* = 50 mm. Setting water as a working fluid whose density, dynamic viscosity at room temperature is 10^3^ kg/m^3^ and 10^−3^ Pa · s, respectively. For simplicity, we replace the elliptical object with an impermeable and nonslip wall at *ξ* = *ξ*_1_ in the computational domain during numerical simulations. To ensure the robustness and accuracy of the simulation, the final number of degrees of freedom (DOFs) was 1,061,832 after laborious simulation exercises, and a multifrontal massively parallel sparse direct solver (MUMPS) was utilized.

As mentioned above, free interferences promise the zero-drag characteristics, hence the drag-reduction effect are exhibited first to examine the validity of the proposed cloaks. To evaluate the drag-reduction performance of elliptical hydrodynamic cloaks, we calculate the drag experienced by the objects both with and without the elliptical hydrodynamic cloaks. Herein, drag (Fig. 1c) is calculated based on the formula^59^ that $\int{\int}_{s}\{\mu [(\frac{\partial u}{\partial y}+\frac{\partial v}{\partial x})+2\frac{\partial u}{\partial x}+(\frac{\partial u}{\partial z}+\frac{\partial w}{\partial x})]+{p}_{x}\}dS$, where *μ* is the dynamic viscosity, *p*_*x*_ denotes the pressure component of the *x* direction; *u*, *v* and *w* represent the velocity components along the *x*, *y*, and *z* direction respectively. *S* signifies the surfaces of the object at *ξ* = *ξ*_1_ for the object-existed case, and of the cloaks at *ξ* = *ξ*_2_ for the cloak case in consideration of the fact that the object and hydrodynamic cloaks should be treated as a whole entity. Additionally, the drag-reduction effect of elliptical hydrodynamic cloaks is calculated by the formula $(1-\frac{{F}_{c}}{{F}_{o}})\times 100 \%$, where *F*_*c*_ (cloak case) and *F*_*o*_ (object-existed case) denote the drag that the objects bear in two cases.

For clarity, we define the object-existed case as only the object exists in the flow field, and the cloak case denotes elliptical hydrodynamic cloaks applied on the object-existed case. Considering the flow channel itself generates flow resistances, the background case, defined as pure background flows without objects serves as the reference point. Apparently, the drag for the object-existed case increases approximately 46–80 times (0.486 N–9.722 N) as *Re* increases from 50 to 1000 (Fig. 1c black line). In contrast, the drag of the cloak case (Fig. 1c red line) tends to be zero (0.006 N–0.211 N), where *Re* ranges from 50 to 1000. This is because there does exist vorticity near the nonslip walls in Hele-Shaw flows (quasi-irrotational flows), which directly affects the near-perfect cloaking effect (see Supplemental Material S.1) based on irrotational flows. Namely, our paradigm is capable of realizing zero-drag characteristics, as well as the resolution of the D’Alembert paradox in viscous potential flows as long as vorticities are eliminated or errors are ignored. The drag for the cloak case increases inconsiderably between *Re*’s of 1000 to 3000, and notably, the corresponding drag-reduction effect exceeds 96% (Fig. 1c cyan line) even when *Re* reaches 3000. The above demonstrates the giant drag-reduction effect that the proposed elliptical hydrodynamic cloaks hold. Indeed, the zero-drag characteristics of the hydrodynamic cloaks do resolve the D’Alembert paradox in viscous potential flows, which has been doubted for a long time. These findings challenge and reframe our conventional comprehension of hydrodynamics regarding the impossibility of zero drag, and present exciting possibilities for drag-reduction technology.

**Fig. 2:**
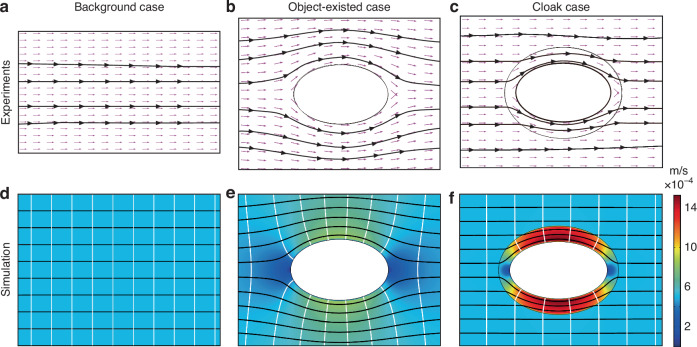
Velocity distributions for the background case (without the elliptical cylinder), object-existed case that only an elliptical cylinder exists in the freestream, and cloak case when hydrodynamic cloaks are applied on the object-existed case. **a**, **b**, **c** Superposition of the experimental streamlines (black lines) with the numerical velocity vectors (magenta arrows) for the three cases. **d**, **e**, **f** Simulation results regarding velocity distributions of three cases superimposed with streamlines (black color) and isobar (white color)

The foregoing observations effectively illustrate zero-drag characteristics of the proposed cloaks, signifying the attainment of the unimpeded interactions (cloaking effect in Fig. 2) between moving objects and the surrounding fluids, as shown in Fig. 2. Generally, the results of experiments [Fig. 2a–c] and simulations [Fig. 2d–f] align well with each other, indicating the validity and correctness of our simulation processes. Unambiguously, it can be observed that the streamlines outside of the cloaked area (regions I and II in Fig. 1b) in the cloak case behave straightly and horizontally (Fig. 2c, f) compared with object-existed case (Fig. 2b, e), and the general flow fields outward the cloaked regions (regions I and II in Fig. 1b) exactly act as that of the background case (Fig. 2a, d). In brief, elliptical hydrodynamic cloaks are capable of restoring the disturbances (Fig. 2b, e) generated by the presence of objects in background flows. Accordingly, the object appears invisible from a hydrodynamic standpoint. Furthermore, it confirms the effectiveness of the proposed thermostatically controlled method in designing hydrodynamic cloaks, as well as the validity of the proposed paradigm.

**Fig. 3:**
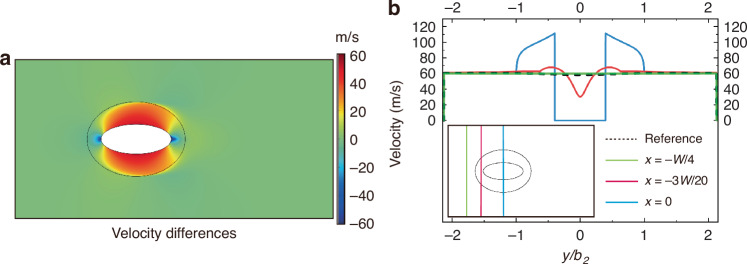
Comparison of velocity distributions at Re = 3000 between the background case (elliptical object absent) and cloak case. **a** Velocity differences distributions between the background case and cloak case. **b** Velocity distributions of cloak case versus *y*/*b*_2_ (*b*_2_ is the minor axis of the cloak) at $x=\frac{-1}{4}W,x=\frac{-3}{20}W$ and *x* = 0. The black dashed line refers to the velocity distributions of the background case

Furthermore, the velocity differences (*Re* = 3000) calculated by the cloak case subtracts from that of the background case displayed in Fig. 3a to demonstrate the cloaking effect quantitatively. Overall, the differences verge on zero outside the cloaked area (regions I and II in Fig. 1b). To further quantitatively verify the cloaking effects of hydrodynamic cloaks, the velocity distributions versus *y*/*b*_2_ (*b*_2_ is the minor axis of the cloak) at $x=\frac{-1}{4}W,x=\frac{-3}{20}W$ and *x* = 0 are exhibited (Fig. 3b). Apparently, the distributions outside the cloaked regions manifest uniform in conformity with that in the background case (black dashed lines). These ulteriorly confirm the near-perfect cloaking effect of elliptical hydrodynamic cloaks and verify the free interferences between the objects and external flows, which firmly supports the drag-free characteristics.

**Fig. 4:**
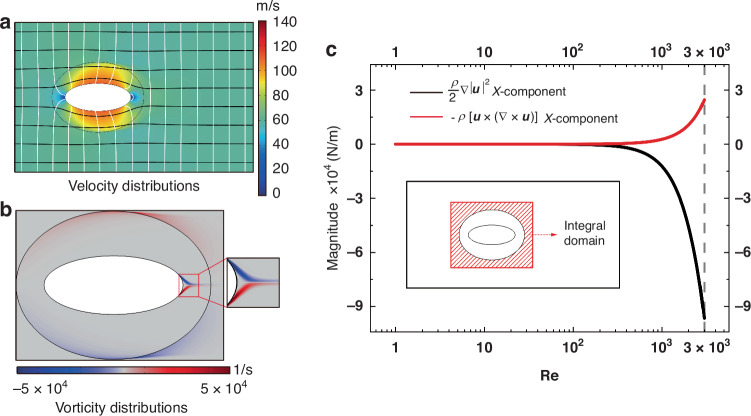
Velocity and vorticity (Ω = ∇ × u) profiles of elliptical hydrodynamic cloaks. **a** Velocity distributions at *Re* = 3000 superimposed with streamlines (black color) and isobars (white color). **b** Vorticity distributions of elliptical hydrodynamic cloaks at *Re* = 3000. **c** Integrated vorticity transport and kinetic-energy gradient magnitudes of elliptical hydrodynamic cloaks against various *Re*’s. The integral domain is the red strip domain on the inset, considering the vorticity generated near the cloaking layer surface

Despite the near-perfect cloaking effect of elliptical hydrodynamic cloaks, we do find that the velocity differences (Fig. 3a) in region III (Fig. 1b), especially the area near the cloaked regions, fluctuate around zero, and drag in cloak case vary under higher *Re*’s (Fig. 1c). Therefore, to investigate the mechanism behind these phenomena, the velocity (Fig. 4a) and vorticity (Fig. 4b) distributions of elliptical hydrodynamic cloaks at a *Re* of 3000 are analyzed. At *Re* = 3000, a slight curvature of the streamlines and isobars near the surface of the cloak can be observed (Fig. 4a). This phenomenon indicates a degradation of the cloaking effect with the simultaneous increase of the drag in the cloak case, where the drag-reduction effect is slightly weakened (96.12%). The key to understanding this phenomenon lies in the vorticity increments in viscous potential flows when the objects exist. Although viscous potential flows behave like ideal flows, the introduction of nonslip walls of the objects, and the velocity differences between the cloaking layer (region II in Fig. 1b) and background (region III in Fig. 1b) do induce the vorticity (Fig. 4b) with the increase of *Re*, which affects the cloaking effect eventually.

According to the theoretical analysis, the convective term in the momentum transport equation $\rho {\boldsymbol{u}}\cdot \nabla {\boldsymbol{u}}=\rho [\frac{1}{2}\nabla | {\boldsymbol{u}}{| }^{2}-{\boldsymbol{u}}\times (\nabla \times {\boldsymbol{u}})]$ is decomposed into two terms, which include the kinetic-energy gradient term $\left(\frac{\rho }{2}\nabla | {\boldsymbol{u}}{| }^{2}\right)$ and vorticity transport term [−*ρ****u*** × (∇ × ***u***)]. In theoretical design, we ignore the vorticity transport term considering the irrotational pattern of the viscous potential flows and then transform the momentum transport equation into a Laplace-like equation serving as the foundation for hydrodynamic zero-drag cloaks with the analytical solution.

To analyze the effect of the vorticity transport term and the kinetic-energy gradient term on cloaking effects, we will investigate the evolutionary characteristics of these two terms. Because the flow is oriented along the main axis (*x*-axis) of the elliptical object, it yields the vorticity transport term and kinetic-energy gradient term of the Y-component much smaller than that of the X-component, allowing the Y-component terms to be negligible (see Supplemental Material S.3). As shown in Fig. 3c, at *Re* values below 1000, the two X-components fall below very small, and the vorticity transport term is relatively negligibly smaller than the kinetic-energy gradient term (see Supplemental Material S.3). However, the vorticity transport rises progressively with increasing *Re* and can no longer be simply ignored compared to the kinetic-energy gradient, which ultimately hinders the realization of drag-reduction and cloaking effects.

Accordingly, it can be defined that the antagonism between the kinetic-energy gradient and vorticity transport determines the rationality of Laplace-like transformation for the momentum transport equation, which identifies the key factor to design the hydrodynamic cloaks with the analytical solution. Namely, when the vorticity transport term remains too insignificant to count compared with the kinetic-energy gradient term, the cloaking effect and the resolution of the D’Alembert paradox in viscous potential flows are guaranteed for hydrodynamic zero-drag cloaks. Furthermore, it also enlightens that our paradigm not only applies to the viscous potential flows but to any real fluids as long as the vorticity among the flow fields is eliminated or the vorticity transport is negligibly overwhelmed by the kinetic-energy gradient. Finally, it implies that hydrodynamic cloaks might be an option to achieve Kelvin-Helmholtz hydrodynamic instability^60^ where vorticity deconstruction is a key factor to understand Kelvin-Helmholtz instability^61^, and a perspective to understand and study the lift^62,63^.

Although the design of the hydrodynamic zero-drag cloak proved to be successful, the proposed paradigm has not realized the hydrodynamic cloaks over *Re* of 3000 and is confined to laminar flows. In the experiment, a more effective method to realize higher-*Re* cloaking effects should be further explored. To overcome these challenges, comprehensive studies for higher-*Re* laminar flows and turbulent flows are required by factoring in the vorticity controlling and other turbulence-related terms. In addition, interdisciplinary research, such as optofluidics, magnetohydrodynamics, electroosmotic flows, and others, should be considered to extend the attainment of hydrodynamic cloaks under higher *Re*’s.

## Conclusion

A paradigm for on-demand zero-drag hydrodynamic cloaks has been provided to resolve the D’Alembert paradox in viscous potential flows. By simplifying and transforming the Navier-Stokes equations into Laplace-like equations, hydrodynamic zero-drag cloaks with the analytical solution are obtained, thus realizing the zero-drag characteristics over a large-*Re* range. This paradigm is experimentally validated by the thermostatically controlled method. Numerical results show that proposed hydrodynamic cloaks achieve zero-drag characteristics when *Re*’s remain under 1000, and continue to exhibit excellent drag-reduction effect even with the increase of *Re* up to 3000. Moreover, hydrodynamic cloaks can switch on and off at will, thus ensuring accurate flow manipulations. These findings offer promising possibilities for drag-free technology, offering valuable insights into microfluidics, biofluidics, and the manipulation of hypervelocity transportation travels hyperloop, among others. Ulteriorly, our research reveals that the vorticity transport directly impacts the performance of the drag-reduction effect and the cloaking effect. This insight suggests that vorticity controlling could be a potential method for designing hydrodynamic zero-drag cloaks, and it may become a critical factor in realizing hydrodynamic zero-drag cloaks under higher *Re*’s, even in turbulent flows.

## Supplementary information

Supplemental Material of the Main Manuscript

Supplementary video for on and off state of hydrodynamic cloak

## Data Availability

The data that support the findings of this study are available from the corresponding author upon reasonable request.
