# Supplementary material for: On-demand zero-drag hydrodynamic cloaks resolve D'Alembert paradox in viscous potential flows
Source: Microsyst Nanoeng. 2024 Dec 12;10:188. doi: 10.1038/s41378-024-00824-z (PMC11638266; doi:10.1038/s41378-024-00824-z)
Supplement: Supplementary file 1 — Supplemental Material of the Main Manuscript [file 41378_2024_824_MOESM1_ESM.pdf]

Supplemental Material

On-Demand Zero-Drag Hydrodynamic Cloaks for High Reynolds Numbers Resolve D'Alembert Paradox

Neng-Zhi Yao,[1] Bin Wang,[1, *] Hao Wang,[1] Chen-Long Wu,[1] Tien-Mo Shih,[2] and Xuesheng Wang[1, *]

[1]School of Mechanical and Power Engineering, East China University of Science and Technology, Shanghai, China.
[2]Department of Mechanical Engineering, University of California, Berkeley, CA, USA.

CONTENTS

S.1. Solutions for hydrodynamic zero-drag cloak parameters 1
S.2. Experimental procedures 4
S.3. Comparison of kinetic energy and vorticity transport against various *Re*'s 7
References 7

S.1. SOLUTIONS FOR HYDRODYNAMIC ZERO-DRAG CLOAK PARAMETERS

Considering the incompressibility, continuity equation and momentum transport equation can be expressed as

$$\nabla \cdot \boldsymbol{u} = 0, \tag{S 1}$$

$$\rho \boldsymbol{u} \cdot \nabla \boldsymbol{u} + \nabla p = \mu \nabla^2 \boldsymbol{u}. \tag{S 2}$$

For viscous potential flows that behave irrotationality, the velocity potential can be introduced, i.e., $\nabla \varphi = \boldsymbol{u}$, and then Eq. (S1) converts to

$$\nabla \cdot \nabla \varphi = 0, \tag{S 3}$$

For convective term in Eq. (S2),

$$\rho \boldsymbol{u} \cdot \nabla \boldsymbol{u} = \rho \left[\frac{1}{2} \nabla |\boldsymbol{u}|^2 - \boldsymbol{u} \times (\nabla \times \boldsymbol{u}) \right]$$

For irrotational flows, Eq. (S2) can be simplified into

$$\mu^{-1}\left(\frac{1}{2}\rho\nabla|\boldsymbol{u}|^2+\nabla p\right)=\nabla^2\boldsymbol{u}$$

Similarly, for diffusion term in Eq. (S2)

$$\nabla^2\boldsymbol{u}=\nabla(\nabla\cdot\boldsymbol{u})-\nabla\times(\nabla\times\boldsymbol{u})$$

Based on Eq. (S1), and taking divergence on both sides, it further can be simplified to

$$\nabla\cdot(\nabla^2\boldsymbol{u})=\nabla\cdot[\nabla\times(\nabla\times\boldsymbol{u})]=0$$

Ultimately, Eq. (S2) is transformed into

$$\nabla\cdot(\mu^{-1}\nabla E)=0, \tag{S 4}$$

where E can be perceived as the mechanical energy density with unit J/m^3.

The analytical solution of Eq. (S4) under elliptical coordinator system (ξ, η) can be generally expressed as

$$\begin{aligned}\Theta_{n,i}=A^i\cosh(n\xi)\cos(n\eta)+B^i\cosh(n\xi)\sin(n\eta)+\\ C^i\sinh(n\xi)\cos(n\eta)+D^i\sinh(n\xi)\sin(n\eta),\end{aligned} \tag{S 5}$$

where $\Theta_{n,i}$ symbolically denotes the n_{th} term of region i in the general solution, and coefficients A^i, B^i, C^i and D^i with superscript (i = I, II, III) are determined by given boundary and interface conditions.

In accordance with the physical fields studied, boundary conditions and finiteness of the geometric model, n = 1. Hence, the expressions of Θ_i can be written as

$$\Theta_{\mathrm{I}}=[A^{\mathrm{I}}\cosh(\xi)+B^{\mathrm{I}}\sinh(\xi)]\cos(\eta), \tag{S 6}$$

$$\Theta_{\mathrm{II}}=[A^{\mathrm{II}}\cosh(\xi)+B^{\mathrm{II}}\sinh(\xi)]\cos(\eta), \tag{S 7}$$

$$\Theta_{\mathrm{III}}=A^{\mathrm{III}}\cosh(\xi)\cos(\eta). \tag{S 8}$$

Note Θ_i represents solutions in each region, that is $i=1$ indicates the objects $(\xi<\xi_1)$, $i=\mathrm{II}$ indicates the hydrodynamic cloak $(\xi_1\le\xi\le\xi_2)$, and $i=\mathrm{III}$ indicates the background $(\xi>\xi_2)$. According to the interface conditions as follows

$$\begin{cases} \Theta_{\mathrm{I}}|_{\xi=\xi_1} = \Theta_{\mathrm{II}}|_{\xi=\xi_1} \\ \zeta_{\mathrm{I}} \dfrac{\partial \Theta_{\mathrm{I}}}{\partial \xi}|_{\xi=\xi_1} = \zeta_{\mathrm{II}} \dfrac{\partial \Theta_{\mathrm{II}}}{\partial \xi}|_{\xi=\xi_1} \\ \Theta_{\mathrm{II}}|_{\xi=\xi_2} = \Theta_{\mathrm{III}}|_{\xi=\xi_2} \\ \zeta_{\mathrm{II}} \dfrac{\partial \Theta_{\mathrm{II}}}{\partial \xi}|_{\xi=\xi_2} = \zeta_{\mathrm{III}} \dfrac{\partial \Theta_{\mathrm{III}}}{\partial \xi}|_{\xi=\xi_2} \end{cases} \quad \text{(S 9)}$$

By substituting Eq. (S9) into Eqs. (S6-S8), the relationship among the dynamic viscosity in different regions can be obtained as

$$\mu_b = \frac{(\mu_c - \mu_h)\tanh(\xi_2) + \mu_h \coth(\xi_1) - \mu_c \tanh(\xi_1)}{(\mu_c - \mu_h)\coth(\xi_2) + \mu_h \coth(\xi_1) - \mu_c \tanh(\xi_1)} \mu_c \quad \text{(S 10)}$$

where μ_b and μ_c denote the dynamic viscosity in background (region III in Fig. 1B) and hydrodynamic cloak (region II in Fig. 1B), μ_h signifies the effective dynamic viscosity of the elliptical cylinder. Because solid walls investigated here are treated as nonslip wall, μ_h then can be perceived infinity and Eq. (S10) simplifies to

$$\mu_c = \frac{-\coth(\xi_2) + \coth(\xi_1)}{-\tanh(\xi_2) + \coth(\xi_1)} \mu_b \quad \text{(S 11)}$$

Boundary conditions of the system are shown below:

The entire system subjects to left-to-right Dirichlet boundary conditions where the incoming velocity of the freestream (region III in Fig. 1B) is $\boldsymbol{U_1}$ along the x direction (main axis of the elliptical cylinder), and P_0 is the pressure of the pressure outlet. The surfaces of all solid walls such as elliptical cylinder (region I) solid wall $\xi = \xi_1$ and walls locate at y = ±0.5 H and z = ± 0.5 D are applied nonslip boundary conditions.

S.2. EXPERIMENTAL PROCEDURES

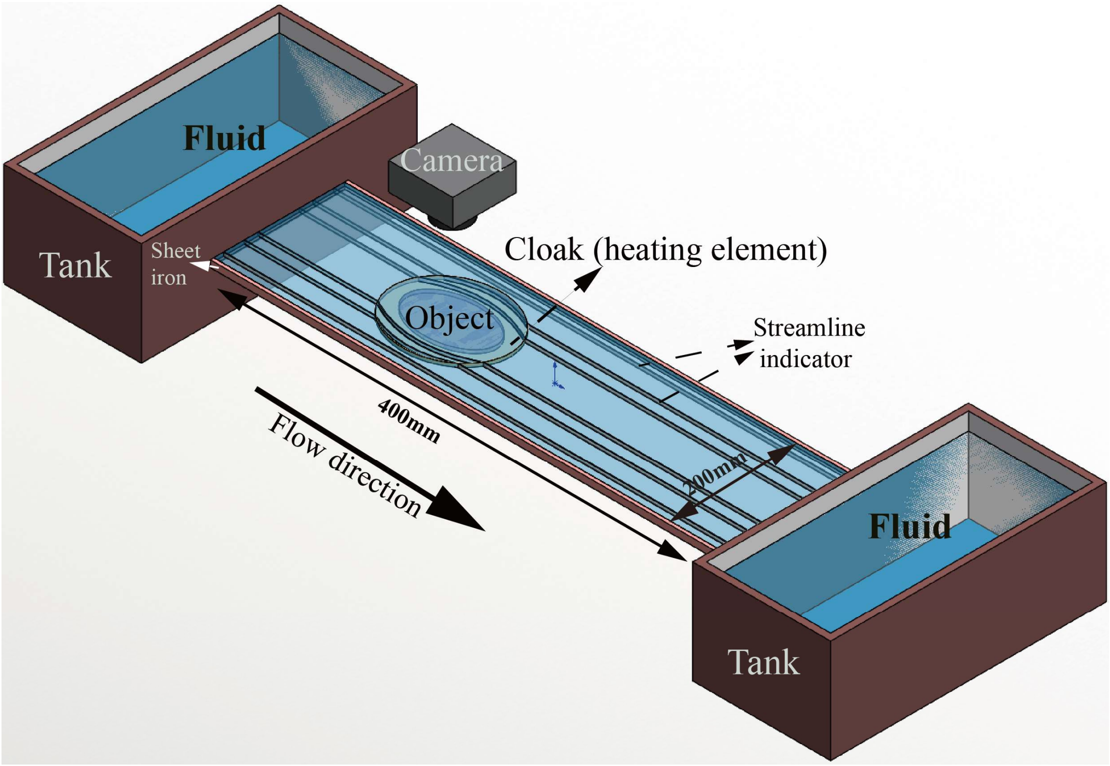

FIG. S1. Schematic diagram of the experimental setup.

A rectangular channel with dimensions 400 mm × 200 mm × 1 mm, features as Hele-Shaw flows (namely viscous potential flows), is placed between two tanks containing glycerin. Two pieces of glass with thickness of 1 mm are placed at the top and bottom of the samples, and the sheet iron whose thickness is 1 mm is placed at each side to enclose the channel. An elliptical object with a major axis $a_1 = 75$ mm and minor axis $b_1 = 46$ mm, along with the height of 1 mm is placed at the front part of the channel. Similarly, the major axis, minor axis and height of the elliptical cloaks are 93 mm, 71 mm and 1 mm respectively. During the experiments, the glycerin is pumped to the tank by peristaltic pump steadily, and then flows into the rectangular channel. Meanwhile, hydrodynamic cloaks composed of heating elements applied on the bottom glass start to heat the glycerin flowing through the cloaked region, which is defined as thermostatically-controlled method. The optimized heating temperature is obtained after laborious simulation exercises in commercial software COMSOL Multiphysics, in conjunction with insights from study [1]. To demonstrate the experiment results visually, streamline indicator that is the glycerin dyed with black paints is injected through four evenly distributed thin rubber tubes by micro-injection pump. The experiments are recorded by a camera from top when the system reaches a steady state.

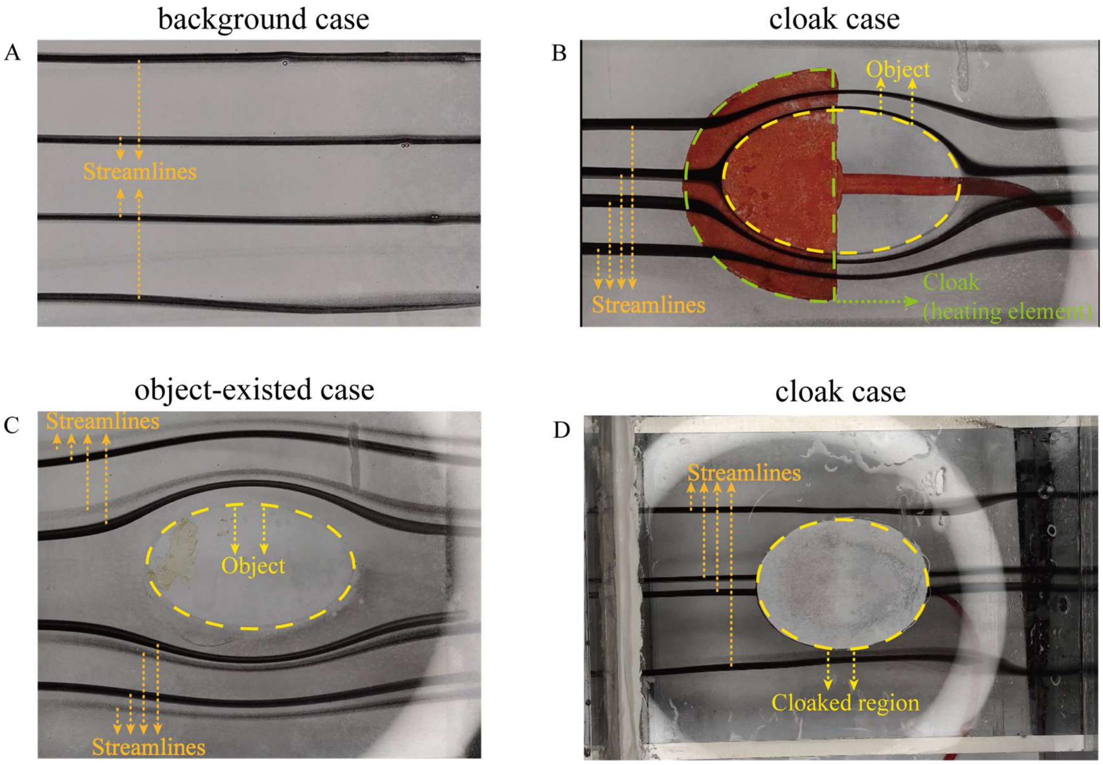

Fig. S2. Actual experimental apparatus and results. (A) Background case that is without the elliptical cylinder existed in the flow field. (B) Cloak case where the streamlines are inside the cloaked region. (C) Object-existed case where an object is present. (D) Cloak case where two of the streamlines are outside the cloaked region. The red semi-ellipse in (B) represents the heating element used to heat fluid, and manipulate the fluid viscosity in the targeted area. The white elliptical region in (D) denotes the cloaked area. Black lines illustrate the streamlines.

To more effectively illustrate the flow state across the entire flow field, we present the streamlines (glycerin dyed with black paint) both inside [Fig. S2(B)] and outside [Fig. S2(D)] the cloaked region [see Fig. S2(D)]. Since the experiments were recorded after the system had reached a steady state and both flow fields in Fig. S2(B, D) achieved the cloaking state, we have combined the streamlines from both figures. Specifically, the streamlines inside and outside the cloaked region are integrated into Fig. 2(C) in the main manuscript to provide a clearer depiction of the experimental results. Additionally, for the scenario where an object is present, we have incorporated six more distinct streamlines from Fig. S2(C) into Fig. 2(B). For background case, the number of the streamlines are maintained four in both Fig. S2(A) and Fig. 2(A). It should be noted that the difference in height of the shot causes the size of the ellipses in Fig. S2 to appear inconsistent, but they are actually the same size.

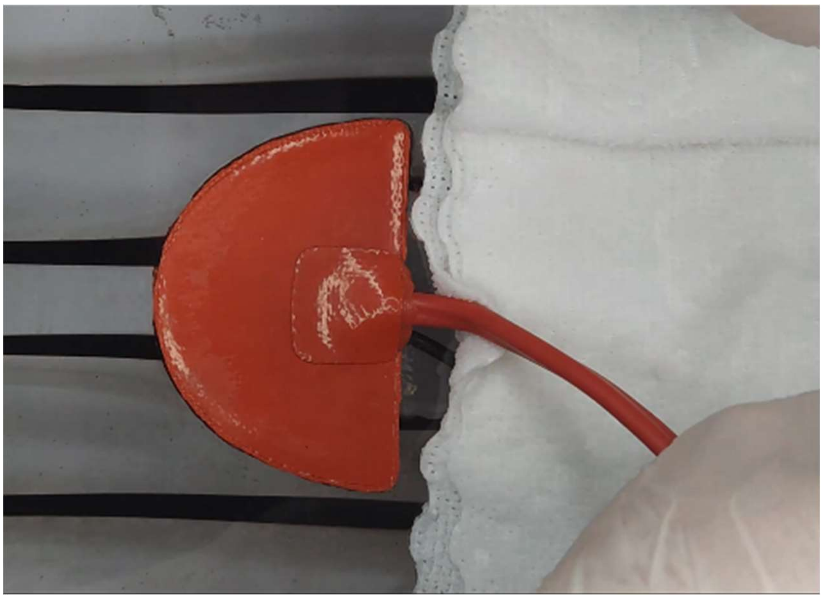

Fig. S3. Cooling measures during the heating process involve using white towels soaked in a mixture of ice water to cool the fluid downstream. The red semi-ellipse represents the heating element used to heat the fluid in the targeted area.

Cooling measures are applied downstream during the heating process [2]. As depicted, white towels soaked in an ice-water mixture are applied to cool the fluid downstream during the experiment (Fig. S3). This effectively mitigates the impact of convective thermal fluxes traveling downstream, thereby maintaining the temperature and viscosity of the background fluid. Once the system reaches the cloaking state, the towels are removed, ensuring that the cooling apparatus does not appear in the final experimental results.

S.3. COMPARISON OF KINETIC ENERGY AND VORTICITY TRANSPORT AGAINST VARIOUS Re's

Table S1. Comparison between X and Y components for gradient of kinetic energy and vorticity transport against various *Re*'s.

Re	Gradient of kinetic energy in X component (N/m)	Gradient of kinetic energy in Y component (N/m)	Vorticity transport in X component (N/m)	Vorticity transport in Y component (N/m)
1	-1.13×10^{-3}	-1.62×10^{-7}	1.92×10^{-4}	3.42×10^{-7}
50	-30.79	1.61×10^{-4}	6.27	-3.86×10^{-4}
100	-126.44	2.42×10^{-4}	26.06	-2.56×10^{-4}
500	-3147.28	1.12×10^{-2}	666.63	-6.47×10^{-4}
1000	-12260.50	1.00×10^{-1}	2673.35	-2.67×10^{-2}
2000	-45850.80	5.97	10706.60	-8.94
3000	-96315.60	21.21	24567.40	-106.20

It can be observed that the gradient of kinetic energy and vorticity transport in Y component is much smaller than that of the X component.

[1] Ferreira A G M, Egas A P V, Fonseca I M A, et al. The viscosity of glycerol. The Journal of Chemical Thermodynamics, 2017, 113: 162-182.

[2] Wang B, Shih T M, Xu L, et al. Intangible hydrodynamic cloaks for convective flows. Physical Review Applied, 2021, 15(3): 034014.
