# Supplementary figures and images for: On-demand zero-drag hydrodynamic cloaks resolve D'Alembert paradox in viscous potential flows
Source: Microsyst Nanoeng. 2024 Dec 12;10:188. doi: 10.1038/s41378-024-00824-z (PMC11638266; doi:10.1038/s41378-024-00824-z)

**Supplementary video for on and off state of hydrodynamic cloak**


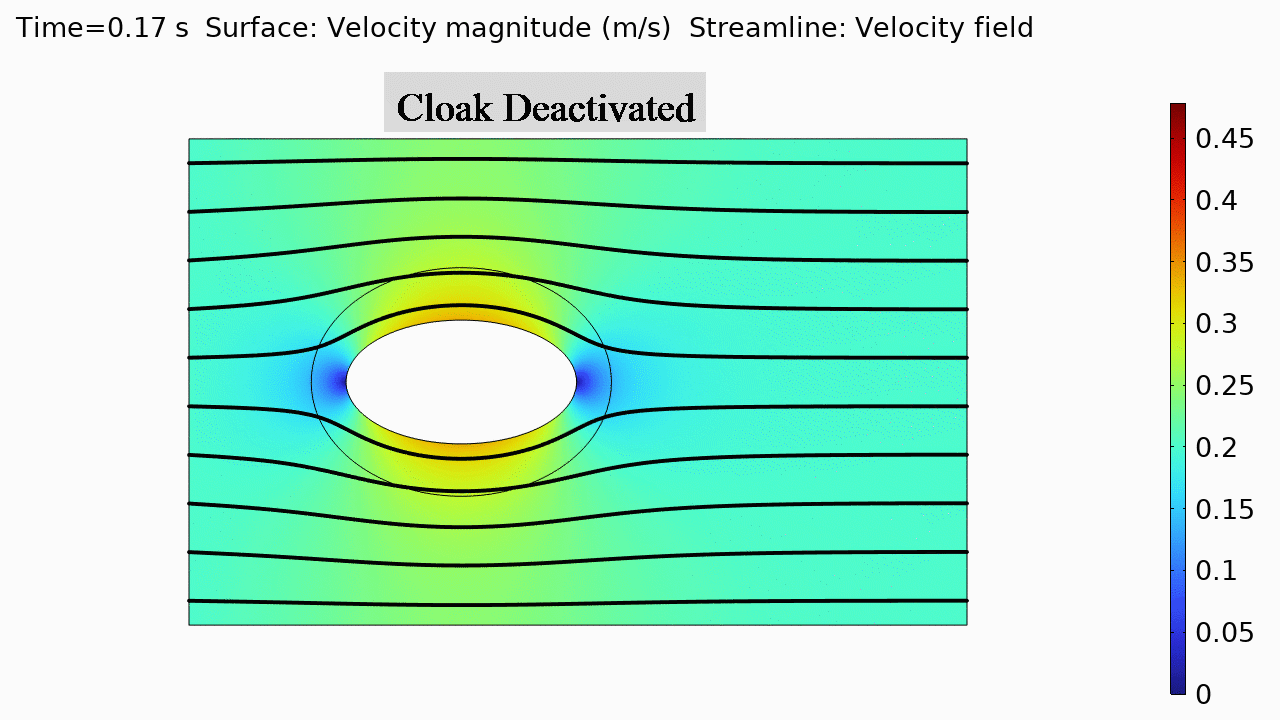

Supplement: Supplementary file 2 — Supplementary video for on and off state of hydrodynamic cloak [file 41378_2024_824_MOESM2_ESM.docx]
